# Nonpharmacological Interventions in Targeting Pain-Related Brain Plasticity

**DOI:** 10.1155/2017/2038573

**Published:** 2017-02-16

**Authors:** Maral Tajerian, J. David Clark

**Affiliations:** ^1^Department of Anesthesiology, Stanford University School of Medicine, Stanford, CA, USA; ^2^Veterans Affairs Palo Alto Health Care System, Palo Alto, CA, USA

## Abstract

Chronic pain is a highly prevalent and debilitating condition that is frequently associated with multiple comorbid psychiatric conditions and functional, biochemical, and anatomical alterations in various brain centers. Due to its widespread and diverse manifestations, chronic pain is often resistant to classical pharmacological treatment paradigms, prompting the search for alternative treatment approaches that are safe and efficacious. The current review will focus on the following themes: attentional and cognitive interventions, the role of global environmental factors, and the effects of exercise and physical rehabilitation in both chronic pain patients and preclinical pain models. The manuscript will discuss not only the analgesic efficacy of these therapies, but also their ability to reverse pain-related brain neuroplasticity. Finally, we will discuss the potential mechanisms of action for each of the interventions.

## 1. Introduction

Chronic pain is a heavy burden for the individual and society affecting 30% of the adult population in the USA [1] and presenting with multiple comorbid psychiatric disorders, including mood alterations [2] and cognitive impairment [3]. With its growing incidence and prevalence, chronic pain is associated with billions of dollars in expenditure related to both therapeutic efforts and costs linked to loss of productivity [4], thus becoming one of our most urgent unmet medical needs. Back pain, headache, and joint pain are some of the most prevalent types of chronic pain [5].

Due to its distressing and unpleasant nature, acute pain serves a protective role against tissue damage. However, under certain circumstances, it can become persistent, eventually presenting as a distinct pathology. One of the pivotal mechanisms that could explain the chronification of pain, as well as its resistance to classical forms of treatment, is the concept of pain centralization, where initial sensory events following trauma can gradually alter the central nervous system (CNS), resulting in amplified pain and/or aberrant pain that exists without peripheral tissue damage or sensitization. In particular, alterations in brain circuitry have been well reported across a wide spectrum of pain conditions, such as complex regional pain syndrome [6, 7], fibromyalgia [8, 9], neuropathic pain [10–13], and migraine [14], thus prompting the quest for treatments that could reset these systems.

Defining the exact circuitry of pain in the brain is complex, mainly because pain is a multidimensional experience that incorporates nociceptive, affective, and cognitive networks. In brief, the dorsal posterior insula, the primary and secondary somatosensory cortices, the anterior insula, the ventrolateral and medial thalamus, the hypothalamus, and the dorsal anterior cingulate cortex (dACC) have been implicated in the nociceptive processing of pain, while limbic systems including the nucleus accumbens, amygdala, and hippocampus could become involved with persistent nociceptive input, eventually engaging prefrontal cortical circuitry [15, 16]. It is important to note, however, that this pain “matrix” is not a static entity but rather a dynamic network that is characterized by specific spatiotemporal neural expression patterns in painful conditions [17].

Current analgesic therapies rely heavily on pharmacological agents and fail in providing relief to a substantial subset of the chronic pain population. Despite the recent advances in understanding the neuroscience of pain and nociception, most drugs fall into a few narrow categories, including opioids which are widely used in patients with moderate to severe chronic pain [18]. With opioids falling increasingly out of favor due to concerns over poor efficacy and abuse, complementary and alternative medicine (CAM) approaches as safe and efficacious replacements or complements to pharmacotherapy are fast gaining popularity [19].

CAM encompasses an array of treatments that fall outside the radius of conventional therapies. It can be used together with conventional therapies (complementary) or in place of conventional therapies (alternative), with most patients receiving a coordinated care regimen that integrates mainstream medicine with complementary approaches to a healthy lifestyle. Despite an initial skepticism towards the analgesic efficacy of such interventions, there is now accumulating evidence regarding the utility of CAM treatments as well as potential underlying mechanisms that could demystify them. To date, there have been few studies directly addressing the effects of CAM analgesic treatments on pain-related neuroplasticity, in large part because the field of brain plasticity that is associated with chronic pain is itself rapidly evolving. This review aims at describing a few of the commonly used, feasible, efficacious, and safe CAM approaches to treating chronic pain and their associated neuroplastic mechanisms in the brain both in chronic pain patients and, where applicable, in preclinical models.

## 2. Attentional and Cognitive Interventions

Attentional and cognitive factors are key modulators in the experience of pain. Below we will discuss some of the most commonly used interventions that have been shown to influence both pain perception and the related brain alterations.

### 2.1. Distraction

This intervention is based on diverting attention from the painful stimulus, instead focusing on cognitively demanding tasks. Multiple functional magnetic resonance imaging (fMRI) and electroencephalography (EEG) studies have shown the efficacy of distraction. For example, in response to the application of a noxious heat stimulus, distraction is associated with decreased pain intensity (as reported by the experimental subject) and decreased activity of the thalamus and insula [20], decreased activity in the somatosensory cortex, and increased activity in the prefrontal areas [21, 22]. Distraction might be particularly efficacious in patients who appear to be excessively attentive to their pain, including high pain catastrophizers [23]. Since distraction analgesia is based on “escaping” the reality of pain, it is therefore important to create distractions that are immersive. As such, we have seen a rise in the use of virtual reality (VR) as an analgesic tool, particularly in acute pain conditions. Unlike classic distraction methods that rely on audiovisual or narrative stimulation, VR relies on the use of a simulated three-dimensional virtual environment with which the patients can interact in seemingly “real” and physical manner, often with the use of a headset or goggles. Due to the simulated nature of these environments, there is a broad range of virtual scenarios that can be presented to the pain patient as a distraction. Much like more classical techniques, VR has been linked to reduced pain ratings as well as decreased brain activity in pain regions such as the ACC, primary and secondary somatosensory cortices, insula, and thalamus [24].

The effects of distraction have been tested in rodent models of pain. Mice were injected with formalin and then placed in a familiar arena containing a novel (nonaversive) object. Despite the fact that the formalin-evoked swelling remained unchanged by the distraction paradigm suggesting a lack of effects on peripheral mechanisms, formalin-injected “distracted” mice spent less time engaged in nocifensive behaviors (licking, biting, shaking, etc.) and demonstrated elevated levels of endogenous cannabinoids in the ventral hippocampus [25].

### 2.2. Mindfulness and Meditation

Mindfulness and meditation practice comprises a host of constructs that focus on mental exercises potentially beneficial in modulating painful stimuli [26]. Unlike distraction, mindfulness relies on being attentive to pain in a nonjudgmental way, with the consensus that openness and acceptance to pain, without attaching any cognitive appraisal to it, diminish pain unpleasantness. In a study of experimental pain in healthy control subjects, those who were given mindfulness and meditation training perceived noxious heat stimulation to be less unpleasant and less intense compared to control subjects [27]. In patients with diverse chronic pain conditions, acceptance and commitment therapy (ACT), emphasizing the willingness to live with the pain rather than expect its full resolution, resulted in reduced pain interference in daily functioning, as well as improvements in measures of anxiety and depression [28]. These differences in pain perception are accompanied by functional and anatomical brain changes. Functionally, expert meditators display low baseline activity in pain-related regions (such as the dorsal anterior insula and anterior mid-cingulate cortex) and the amygdala, in addition to enhanced activity in pain-related regions during painful stimulation [29]. In another study, expert (pain-free) meditators showed lower activity in the mid-cingulate cortex, secondary somatosensory cortex, and insula during the painful stimulus [30]. Anatomically, meditation was shown to be associated with increased gray matter thickness in the secondary somatosensory and dorsal anterior cingulate cortices [31]. These techniques are not only applicable to highly trained and long-term meditation practitioners. In a study employing pain-free subjects, a 4-day mindfulness/meditation training program was sufficient in reducing experimental pain unpleasantness and pain-related activation of the primary somatosensory cortex [27].

### 2.3. Cognitive Behavioral Therapy (CBT)

One of the most common CAM approaches to the treatment of chronic pain is cognitive behavioral therapy (CBT). This psychosocial intervention relies on cognitive and behavioral approaches to maximize coping strategies and minimize unhelpful thoughts and attitudes towards chronic pain. These techniques include, but are not limited to, homework assignment (e.g., keeping a pain journal), relaxation techniques (deep breathing, progressive muscle relaxation, etc.), positive affirmation, relapse prevention, operant behavioral therapy, and biofeedback (using monitoring devices) [32]. Despite the somewhat mixed reports for CBT efficacy [33–36], there is some evidence that it can affect pain-related cortical alterations. For example, in a cohort of patients with painful irritable bowel syndrome, a 10-week CBT course was linked to a reduction in pain and anxiety, in addition to reduced activation in brain regions thought to be involved in the emotional and cognitive modulation of pain [37]. In a cohort of female fibromyalgia patients, a 12-week CBT course was paralleled by improvements in depression and anxiety and increased activation in brain areas involved with executive cognitive control [38]. More recently, Seminowicz et al. reported that, following an 11-week long CBT course, chronic pain patients showed improved clinical outcomes in addition to increased gray matter density in the prefrontal and posterior parietal cortices [39].

## 3. Environmental Influences

Environmental influences play a significant role in the prevalence of chronic pain in humans, with low socioeconomic status being associated with higher prevalence of chronic pain conditions both in childhood and adolescence [40] and in adult populations [41]. In the absence of randomized controlled studies, we cannot discount the possibility that these findings reflect a general link between overall health and socioeconomic disadvantage. Nevertheless, there are a few reports of environmental manipulations modulating the perception of pain. In a study conducted in surgical patients, exposure to natural lighting was associated with diminished analgesic usage [42]. Furthermore, under experimental pain conditions, visual images of natural scenery were able to increase both pain thresholds and pain tolerance in control subjects [43]. It is unclear, however, whether these improvements are due entirely to an enriched environment or perhaps due in part to distraction provided by a novel environment. In contrast to the limited set of data available in human subjects, environmental manipulations in animal models of pain are well-studied. The subsequent paragraph will address the effects of environmental manipulations on reversing or preventing the neuroplastic changes that accompany chronic pain.

Similar to clinical observations, preclinical pain measurements are especially sensitive to environmental factors [44]. Housing conditions have been extensively studied, with the consensus that enriched environments are often associated with diminished pain, with slight variations between different reports. Most enriched environments aim to foster natural rodent behaviors and may include the presence of cagemates, textured bedding, activity wheels, various objects that the animals can interact with, and marbles and other similar items buried in the bedding. The effects of such enriched environments have been reported in multiple models: for example, in a rat model of inflammation, environmental enrichment (EE) was associated with reduced thermal, but not mechanical hyperalgesia [45], and in a model of chronic pain following spinal cord injury, rats housed in enriched environments after injury showed a rapid normalization of mechanical allodynia in addition to improved gross locomotor performance [46]. Similarly, in a mouse model of peripheral neuropathy, EE that was administered 3 months after injury attenuated mechanical and cold allodynia [47]. In addition to a stand-alone therapy, EE synergizes with pharmacological agents in targeting experimental pain. For example, the antinociceptive effects (tail withdrawal test) of both mu [48] and kappa opioids [49] are enhanced in EE rats. It is noteworthy that most EE paradigms rely on both social (cagemates) and physical (cage dimensions, inanimate objects within the cage, etc.) enrichment, with physical enrichment having a larger experimental (antiallodynic) effect under certain conditions [50].

Despite the abundance of studies pertaining to behavioral plasticity after EE in preclinical models of pain, there is a surprising paucity of data regarding the underlying brain neuroplasticity. A study from our group investigated the effects of EE on pain-associated aberrant epigenetic modifications in the prefrontal cortex (PFC) of the mouse and showed that global hypomethylation in the PFC, an epigenetic signature of chronic pain in the brain, is absent in the EE group, although the specific genes regulated by methylation in EE were not identified [51]. In another study, Terada et al. demonstrated that EE-induced hippocampal neurogenesis is hampered in chronic pain, although the effects of EE on pain measures were not considered [52]. Finally, in a model of peripheral neuropathy, Norman et al. showed social isolation to be associated with depression and IL1-*β* upregulation in the frontal cortex, both of which were reversed by central oxytocin administration [53]. These results provide preliminary molecular and biochemical links between EE and pain-related brain neuroplasticity. While much of the published literature focuses on EE in chronic pain conditions and its role in ameliorating allodynia and hyperalgesia, there is some evidence that EE could increase nocifensive responses to inflammation. In a mouse model of formalin-induced inflammation, enriched animals demonstrated increased licking as well as increased response to a “safety” signal (dimly lit quarters). These behavioral changes were paralleled with increased plasticity in the ACC [54] and could reflect emotions of fear and safety that are associated with pain.

## 4. Exercise and Physical Rehabilitation

The chronic pain patient population is highly heterogeneous, with a wide range of physical abilities and levels of disability. Nonetheless, physical activity is highly recommended for most patients, with results being comparable to the use of nonsteroidal anti-inflammatory drugs (NSAIDS) and simple analgesics [55–58]. Below we review some evidence supporting the role of exercise in improving pain outcomes, as well as associated brain neuroplastic phenomena, in preclinical and clinical pain populations.

Clinically, chronic pain is often linked with motor disturbances, potentially due to physiological impairment, limb immobilization, or kinesiophobia. Such motor disturbances are associated with alterations in cortical networks perceiving and regulating motor function [59]. It is therefore not surprising that, in addition to neuroplasticity in the “pain matrix,” multiple pain conditions are associated with alterations in the motor cortex, particularly if motor disability is comorbid with the pain. For instance, in patients with chronic low back pain, decreased excitability in the primary motor cortex (M1) [60] and diminished intracortical motor inhibition in M1 circuits [61] have been reported. It is therefore plausible that motor training and physical rehabilitation might be considered as therapeutic options. Indeed, in a study conducted by Tsao et al., low back pain patients exhibited a delay in the postural activation of deep abdominal muscles in addition to abnormal motor representation of this muscle group in the motor cortex, parameters that were both normalized following motor skill training of the muscle group [62]. Unfortunately, self-paced exercise failed to elicit similar improvements [62], suggesting the need for targeted physical rehabilitation.

The antinociceptive and analgesic effects of physical exercise have been shown in rodent models of pain as well, both as prophylactic [63] and therapeutic [64] interventions. While exercise has similarities to EE, it is nonetheless distinct since it not only fosters “natural” rodent behaviors, but actively aims to model aspects of physical rehabilitation commonly employed in the clinic. One of the few studies that addresses the topic of brain plasticity after exercise in animal models of pain comes from Sluka et al. where regular physical activity was shown to prevent the development of chronic muscle pain and to downregulate the phosphorylation of the glutamate receptor NMDA-R1 in the rostral ventromedial medulla, in the absence of any effects on acute nociception [65].

When discussing the “desirable” effects (antidepressive, antiallodynic, analgesic, antinociceptive, etc.) of physical exercise, we must distinguish between voluntary and forced activity. In rodent studies, it appears that the positive outcomes of innately driven exercise could be reversed if the animal subjects are forced to exercise. As such, forced exercise is associated with stress-induced hyperalgesia [66] (thus negating the desirable effects of exercise) or stress-induced analgesia [67] (thereby obscuring the interpretation of the acquired data). These differential effects are also paralleled by brain alterations: for example, forced swimming in rats is associated with increased hyperalgesia after peripheral inflammation, in addition to biochemical and epigenetic marks of plasticity in the insular cortex [68]. It is possible that a similar scenario exists in pain patients as well: those who choose to lead an active lifestyle might benefit the most from it, while those who view it as an unpleasant obligation might profit from the addition of CBT or other intervention that changes their mindset regarding physical exercise.

## 5. Potential Mechanisms of Action

The interventions reviewed in this manuscript affect multiple organ systems in the body, and as such, it is difficult to trace the exact mechanisms by which it can alter pain-related brain plasticity. Below, we describe several potential routes by which CAM therapies can play a part.

### 5.1. Blood-Brain Barrier (BBB) Permeability

BBB compromise has been described in both preclinical [69, 70] and clinical [71, 72] pain conditions and is an attractive candidate for linking peripheral changes following a painful injury to behavioral changes associated with brain plasticity [73]. One possible mechanism by which exercise could prevent some of the maladaptive neuroplasticity observed in chronic pain is limiting BBB permeability after peripheral injury. For instance, data from an experimental model of autoimmune encephalomyelitis shows physical exercise to be associated with the reestablishment of tight junctions and the partial restoration of the BBB [74]. This is particularly relevant to pain-associated comorbidities: in both preclinical models and patients with chronic pelvic pain, pain and depression are associated with elevated levels of prostate-derived cytokines in the cerebrospinal fluid [75], suggesting a BBB breach.

### 5.2. Normalization of Endogenous Neuroplasticity and Neurogenesis

In addition to alterations in motor areas, motor deficits that often parallel chronic pain can also hamper endogenous neurogenesis, in both mice and humans [76]. Furthermore, chronic pain itself is associated with altered neurogenesis, despite the presence of conflicting studies regarding the relationship between the two [77]. It is therefore possible that physical exercise and EE can restore some of endogenous neurorestoration, potentially through antineuroinflammatory mechanisms [78, 79], thereby altering the processing of nociceptive signals. Furthermore, both exercise and EE can have beneficial effects on anxiety and memory deficits that often coexist with pain. Clinical studies show that exercise induces neuroprotection and synaptic strengthening and improves cognitive function as well as motor control in Parkinson's disease [80]; preclinically, EE paired with exercise stimulates neurogenesis in transgenic mice with impaired neurogenesis and reverses the observed memory deficits [81] and, in WT mice, this exercise/EE paradigm reduces anxiety and improves memory. Additional observations demonstrate that EE/exercise modulates multiple gene targets with known involvement in synaptic plasticity [82].

### 5.3. Ascending Control of Nociceptive Signals

Nociceptive information travels from peripheral nociceptors and dorsal horn to the thalamus (spinothalamic tract) and brainstem and medulla (spinoreticular and spinomesencephalic tracts). Mindfulness therapy and other similar cognitive interventions could interfere with the ascending nociceptive signal, since they rely on a “bottom-up” approach that focuses on the pain sensation without appraising it in any way. To date, there is some conflicting evidence for thalamic modulation in CAM therapies.

On one hand, there is evidence for thalamic activation in CAM-related pain amelioration. For instance, mindfulness practitioners have lower pain sensitivity in addition to increased thalamic activation and decreased connectivity between cognitive (e.g., dorsolateral PFC [dlPFC]) and pain-related (e.g., ACC) cortices [83]. Additionally, it is possible that thalamic activation during exercise could hinder the relay of nociceptive signals. This hypothesis is supported by both the robust and direct anatomic connections between the motor cortex and the thalamus, by clinical data showing effective neuropathic pain relief by motor cortex stimulation [84], and by preclinical data where chronic exercise was paralleled by an increased activation of the cerebellar-thalamic-cortical circuit in rats [85], thus providing additional incentive for ongoing physical activity in pain patients. For those patients with reduced mobility, ascending noxious signals can be modulated via guided imagery. For instance, motor imagery under hypnotic trance results in thalamic activation [86].

On the other hand, some studies have found thalamic inhibition to be linked with decreased nociception. In a study conducted by Pagano et al., motor cortex stimulation (without any physical exercise) was shown to result in increased nociceptive thresholds and the inhibition of thalamic hyperactivity in naïve rats [87].

The seeming discrepancy between these sets of findings could be due to multiple factors, including the pain-free versus chronic pain state of the subjects (thalamic activity varies significantly between these two groups [88]), the type of intervention, and the alternate mechanisms that could be at play. For example, it is possible that motor imagery is efficacious, at last in part, through its ability to function as a distraction agent. Finally, the thalamus is part of a complex set of circuits and pathways that constitute the pain matrix. As such, it is an oversimplification to assume thalamic activation alone as a proxy for pain relay and processing.

### 5.4. Descending Modulation of Pain

In addition to controlling ascending signals, the brain exerts control over nociception via a descending brain network that encompasses the dlPFC, ACC, insula, hypothalamus, rostral ventromedial medulla (RVM), and the periaqueductal gray (PAG) [89]. These “top-down” regulators may be disrupted in chronic pain conditions and may be rectified by nonpharmacological means: For example, distraction, by the virtue of being a “top-down” pain regulator, could possibly act through the modulation of descending pain [22]. It is even arguable that classic descending noxious inhibitory control (DNIC) paradigms are efficacious due to the element of distraction (distracting one type of pain by another) [90]. By the same token, descending pain modulation is a likely mechanism of action for CBT as well, where improved clinical outcomes could be due to enhanced top-down control of pain (pain modulation) and altered experience of noxious stimuli (pain perception), as evidenced by CBT-associated increase in gray matter density in the dlPFC and posterior parietal cortex [39]. Finally, this top-down regulation of pain can be modulated through exercise: in a study conducted in a cohort of fibromyalgia patients (compared to control subjects), a brief bout of exercise was shown to modulate pain and stimulate the anterior insula and the dlPFC [91].

Serotonergic, dopaminergic, and noradrenergic pathways are all involved in modulating the facilitatory and inhibitory pain drives. In brief, serotonin (5-HT) and dopamine (D) can exert both pro- and antinociceptive effects, depending on the type of pain and the expression of its receptors (5-HT1, D2, and D3 being antinociceptive and 5-HT2, 5-HT3, and D1 being pronociceptive), and noradrenergic pathways have been shown to be mainly antinociceptive (for review, please see [92]). The balance between these various drives is altered in chronic pain. For instance, nerve injury is accompanied by an overall enhancement of the descending 5-HT facilitatory drive [93] and chronic peripheral inflammation is paralleled by increased activity in the descending dopaminergic pathway [94]. The pain-related alterations in these monoaminergic pathways can be modulated by physical exercise. In rodents, peripheral neuropathy was ameliorated by low intensity aerobic exercise and was associated with increased 5-HT and 5-HT receptor content, reduced 5-HT turnover, and decreased proinflammatory cytokine levels in the brainstem [95].

### 5.5. Opioid Regulation

Data from animal studies show the involvement of EE is regulating opioids, with somewhat conflicting results. While rodent data shows that EE is commonly associated with activated opioid signaling [96], data from porcine subjects shows that enriched housing environments are associated with decreased expression of opioid receptors in the amygdala [97].

In general, exercise is associated with increased endogenous opioids in healthy subjects [98]. In the chronic pain population, this link is less clear: On one hand, there is evidence of dysfunctional regulation of central (hypothalamus) and peripheral (pituitary) endogenous opioids following acute bouts of exercise [99]; on the other, motor cortex stimulation in chronic pain patients was linked to pain relief as well as the release of endogenous opioids in the anterior middle cingulate cortex and the PAG [100]. Preclinically, physical activity is commonly associated with increased endogenous opioid peptides, and increased *μ*-opioid receptors have been reported in the rat hippocampus following acute and chronic exercise [101]. Moreover, rats who were bred for high motivation for voluntary running showed elevated opioidergic signaling in the nucleus accumbens [102], and hyperalgesia following limb immobilization in rats was ameliorated by treadmill exercise and was linked with increased levels of *β*-endorphins in the hypothalamus and midbrain PAG [103].

The role of endogenous opioids in mindfulness/meditation is less clear: in a study conducted in healthy meditation practitioners, the analgesic effects of meditation were reversed by the administration of the opioid antagonist naloxone [104]. In contrast, in meditation-naïve healthy participants, a 4-day mindfulness/meditation training protocol resulted in analgesic effects that were naloxone independent [105]. It is therefore possible that the duration of meditation practice is key in recruiting various neuroplastic mechanisms for pain perception and regulation.

### 5.6. Endocannabinoid Mechanisms

The endocannabinoid system has recently emerged as a potential therapeutic target for multiple chronic pain conditions [106]. Similar to the aforementioned opioid-mediated analgesia, cannabinoid mediated analgesia and antinociception are mediated by brainstem circuits, including the inhibition of GABA release in the PAG and RVM [107]. In chronic pain, there is evidence from rodent studies showing that CB1R, one of the two main cannabinoid receptors, is downregulated in the RVM, with CB2R playing a compensatory role in GABA modulation [108].

Data from a rat model of formalin-evoked pain shows the endocannabinoid system to be involved in distraction-induced antinociception, where distraction is associated with increased levels of the endogenous ligands in the ventral hippocampus, and the administration of a CB1R antagonist attenuates distraction-induced analgesia [109]. Similarly, both aerobic exercise and resistance training in rats were shown to be associated with increased nociceptive thresholds as well as increased CB1R levels in the PAG [110, 111].

### 5.7. Placebo

Clinically, pain is usually measured as a subjective report and is particularly sensitive to the placebo effect through both opioid and cannabinoid systems [112–114]. However, it would be shortsighted to equate placebo treatments with the administration of an inert substance. Instead, the key to the measurable and physical effects that placebo has may lie in the treatment or care that the patient receives. Viewed in this light, it is plausible that various CAM modalities exert significant placebo effects in part because of expectations of alleviation of pain. Particularly in those patients where CAM is integrated alongside more traditional pharmacotherapy, a “preconditioning” effect could take place, where the pharmacological agent both relieves the pain and boosts the efficacy of the placebo [115], even reaching the extent of overriding the knowledge that the intervention is only a placebo [116].

## 6. Limitations and Future Directions

It is noteworthy that many of the studies reviewed here do not distinguish between the neuroplastic changes that occur indirectly through the amelioration of pain through CAM approaches versus the direct effect of these CAM interventions on the brain. Indeed, many of the described neuroplastic changes may not be unique to pain but could rather serve as a proxy for the plethora of pain-associated comorbidities, including memory deficits, anxiety, and depression. Additionally, much of the reviewed data was collected in pain-free control subjects under experimental pain conditions. These data may not be directly relevant to the chronic pain brain, since pain perception in healthy subjects is radically different from that in chronic pain patients. Finally, despite the multiple reports showing beneficial results of CAM treatments, we lack concrete evidence of their efficacy in different pain conditions, especially since there are preclinical [117] and clinical [118] studies that fail to show any benefits. We anticipate that future research findings both from preclinical studies and from controlled clinical trials will provide us with an improved mechanistic understanding of the efficacy of CAM therapies in the treatment of chronic pain.

## 7. Conclusions

**Figure 1:**
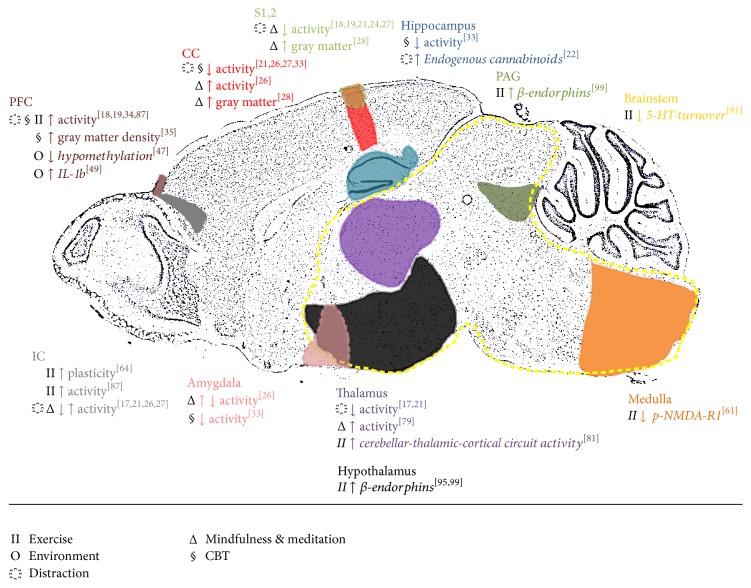
Illustrated summary of key CAM-responsive centers in the pain brain. Text in colors correspond to each painted brain region in that same color. Due to the single sagittal view of the brain, some areas may not be visualized in their entirety or may not be true to scale. CC: cingulate cortex; IC: insular cortex; IL-1b: interleukin 1-beta; PAG: periaqueductal gray; PFC: prefrontal cortex; S1,2: primary and secondary somatosensory cortices; 5-HT: 5-hydroxytryptamine/serotonin. Text in italics refers to findings from rodent studies.

This review summarizes the effects of noninvasive treatments in preventing or reversing pain-related alterations in brain biochemistry, structure, and function in preclinical models as well as chronic pain patients (please refer to Figure 1 for an illustrated summary). The limited efficacy of traditional pharmacotherapy, along with our increased understanding of the mechanisms behind the action of complementary therapies, has led the shift towards a more holistic view of pain treatment, where long-lasting supra-spinal changes are targeted.
